# Supplementary material for: Association between COPD and Stage of Lung Cancer Diagnosis: A Population-Based Study
Source: Curr Oncol. 2023 Jul 5;30(7):6397–410. doi: 10.3390/curroncol30070471 (PMC10377848; doi:10.3390/curroncol30070471)
Supplement: Supplementary file 1 [file curroncol-30-00471-s001.zip › curroncol-2459307-supplementary.pdf]

Association between COPD and stage of lung cancer diagnosis: A population-based study
Butler SJ, Louie AV, Sutradhar R, Paszat L, Brooks D, Gershon AS.

Online Supplement

Table S1: Lung Cancer Morphology Codes

Type of Lung Cancer	Morphology Codes
Non-small cell lung cancer (NSCLC)	
Adenocarcinoma	80503, 80523, 81303, 81403, 81406, 82503, 82513, 82523, 82533, 82543, 82553, 82563, 82573, 82603, 82653, 84803, 84813, 85503, 85513, 85713, 85723, 85743, 85753, 85763
Squamous cell carcinoma	80703, 80713, 80723, 80733, 80743, 80763, 80833, 80843
Large cell carcinoma	80033, 80043, 80123, 80133, 80143, 80303, 80313, 80323, 80343, 80353, 82463
Adenosquamous	85603, 85623
Not Otherwise Specified	80103, 80203, 80463, 81233
Small cell lung cancer (SCLC)	80023, 80413, 80423, 80443, 80453
Unspecified	80003, 80013, 82303

Table S2: Balance in characteristics of individuals with previously diagnosed COPD and without COPD prior to and after inverse propensity score weighting (IPW)

	Unweighted			Weighted		
	**Previously diagnosed COPD** (n=38,894)	**No COPD** (n=39,098)	**SMD**	**Previously diagnosed COPD**	**No COPD**	**SMD**
**Demographics**						
Age, mean (SD)	71.9 (9.4)	68.9 (11.3)	0.284	70.7 (9.5)	70.6 (11.3)	0.012
Sex, % male	51.5%	51.0%	0.010	51.7%	51.5%	0.003
Rurality/Income Quintile						
Rural	16.5%	13.8%	0.077	15.2%	15.0%	0.004
Urban 1 (lowest)	23.3%	18.2%	0.124	20.6%	20.5%	0.002
Urban 2	19.6%	18.8%	0.020	19.4%	19.4%	0.001
Urban 3	15.8%	17.1%	0.034	16.5%	16.4%	0.001
Urban 4	13.4%	16.5%	0.087	14.8%	15.0%	0.003
Urban 5 (highest)	11.4%	15.6%	0.125	13.5%	13.6%	0.004
Immigration Category						
</= 10 years	0.4%	2.2%	0.159	1.1%	1.3%	0.012
Long-term resident (> 10 years)	2.7%	6.6%	0.189	4.5%	4.7%	0.005
Non-immigrant	97.0%	91.2%	0.244	94.3%	94.1%	0.010
**Type of Lung Cancer**						
NSCLC: Adenocarcinoma	35.5%	49.4%	0.281	42.5%	42.9%	0.007
NSCLC: Squamous cell carcinoma	21.9%	13.9%	0.211	17.9%	17.8%	0.003
NSCLC: Large cell carcinoma	2.4%	2.7%	0.017	2.6%	2.6%	0.003
NSCLC: Adenosquamous	0.5%	0.6%	0.010	0.6%	0.6%	0.003
NSCLC: Not otherwise specified	14.2%	14.2%	0.001	14.1%	14.1%	0.002
SCLC	12.7%	10.4%	0.072	11.5%	11.4%	0.003
Unspecified	12.7%	8.8%	0.126	10.8%	10.6%	0.005
**Comorbidities**						
Asthma	26.1%	7.9%	0.485	16.9%	16.2%	0.019
Congestive heart failure	17.7%	7.5%	0.309	12.6%	12.4%	0.006
Dementia	5.5%	3.7%	0.087	4.7%	4.6%	0.001
Diabetes	29.4%	24.8%	0.105	27.3%	26.9%	0.009
Previous pneumonia	32.5%	18.5%	0.321	25.4%	25.0%	0.009
Cancer in the previous 5 years	8.7%	9.1%	0.013	9.0%	9.0%	0.003
**Previous Healthcare Utilization**						
Rate of primary care visits per year						
5 or more	50.6%	34.0%	0.337	42.4%	42.0%	0.008
Between 2 and 5	35.1%	38.1%	0.064	36.8%	36.9%	0.001
2 or less	12.6%	22.3%	0.255	17.4%	17.5%	0.001
0	1.7%	5.6%	0.208	3.3%	3.6%	0.016
Specialist care	49.7%	36.4%	0.269	43.4%	43.0%	0.009

Abbreviations: NSCLC = Non-small cell lung cancer, SCLC = small cell lung cancer, SMD = standardized mean difference, SD=standard deviation

Table S3: Balance in characteristics of individuals with undiagnosed COPD and without COPD prior to and after inverse propensity score weighting (IPW)

	Unweighted			Weighted		
	**Undiagnosed COPD** (n=8,843)	**No COPD** (n=39,098)	**SMD**	**Undiagnosed COPD**	**No COPD**	**SMD**
**Demographics**						
Age, mean (SD)	68.7 (9.9)	68.9 (11.3)	0.019	68.9 (10.0)	68.9 (11.3)	0.001
Sex, % male	54.7%	51.0%	0.075	52.4%	51.7%	0.013
Rurality/Income Quintile						
Rural	15.5%	13.8%	0.051	14.2%	14.1%	0.002
Urban 1 (lowest)	21.3%	18.2%	0.079	18.8%	18.8%	0.001
Urban 2	20.3%	18.8%	0.037	19.1%	19.1%	0
Urban 3	15.7%	17.1%	0.036	16.8%	16.8%	0
Urban 4	14.4%	16.5%	0.058	16.0%	16.1%	0.003
Urban 5 (highest)	12.8%	15.6%	0.081	15.1%	15.1%	0
Immigration Category						
</= 10 years	1.5%	2.2%	0.048	1.9%	2.0%	0.009
Long-term resident (> 10 years)	4.2%	6.6%	0.103	6.2%	6.2%	0
Non-immigrant	94.4%	91.2%	0.115	91.9%	91.8%	0.004
**Type of Lung Cancer**						
NSCLC: Adenocarcinoma	42.2%	49.4%	0.144	47.8%	48.1%	0.004
NSCLC: Squamous cell carcinoma	20.5%	13.9%	0.185	15.2%	15.1%	0.002
NSCLC: Large cell carcinoma	2.6%	2.7%	0.009	2.7%	2.7%	0
NSCLC: Adenosquamous	0.7%	0.6%	0.010	0.6%	0.6%	0
NSCLC: Not otherwise specified	13.6%	14.2%	0.019	14.1%	14.1%	0
SCLC	11.7%	10.4%	0.041	10.8%	10.6%	0.006
Unspecified	8.8%	8.8%	0.001	8.7%	8.8%	0.002
**Comorbidities**						
Asthma	9.9%	7.9%	0.072	8.3%	8.3%	0.002
Congestive heart failure	9.7%	7.5%	0.084	7.9%	7.9%	0
Dementia	2.8%	3.7%	0.049	3.5%	3.5%	0.001
Diabetes	23.3%	24.8%	0.034	2.5%	2.5%	0.002
Previous pneumonia	24.3%	18.5%	0.146	19.6%	19.5%	0.001
Cancer in the previous 5 years	6.6%	9.1%	0.086	8.4%	8.6%	0.006
**Previous Healthcare Utilization**						
Rate of primary care visits per year						
5 or more	31.3%	34.0%	0.056	33.5%	33.5%	0.001
Between 2 and 5	37.0%	38.1%	0.023	37.9%	37.9%	0
2 or less	24.9%	22.3%	0.062	22.8%	22.8%	0.001
0	6.7%	5.6%	0.047	5.8%	5.8%	0.003
Specialist care	34.7%	36.4%	0.035	36.1%	36.1%	0.001

Abbreviations: NSCLC = Non-small cell lung cancer, SCLC = small cell lung cancer, SMD = standardized mean difference, SD=standard deviation

Table S4: Balance in characteristics of individuals with previously diagnosed and undiagnosed COPD prior to and after inverse propensity score weighting (IPW)

	Unweighted			Weighted		
	**Undiagnosed COPD** (n=8,843)	**Previously diagnosed COPD** (n=38,894)	**SMD**	**Undiagnosed COPD**	**No COPD**	**SMD**
**Demographics**						
Age, mean (SD)	71.9 (9.4)	71.9 (9.4)	0.334	71.6 (10.0)	71.3 (9.4)	0.026
Sex, % male	51.5%	51.5%	0.065	52.2%	52.1%	0.004
Rurality/Income Quintile						
Rural	16.5%	16.5%	0.027	16.2%	16.3%	0.003
Urban 1 (lowest)	23.3%	23.3%	0.046	22.5%	22.9%	0.008
Urban 2	19.6%	19.6%	0.017	19.9%	19.8%	0.004
Urban 3	15.8%	15.8%	0.002	16.2%	15.8%	0.010
Urban 4	13.4%	13.4%	0.029	13.6%	13.6%	0
Urban 5 (highest)	11.4%	11.4%	0.043	11.6%	11.6%	0.002
Immigration Category						
</= 10 years	0.4%	0.4%	0.145	0.5%	0.6%	0.001
Long-term resident (> 10 years)	2.7%	2.7%	0.089	2.9%	3.0%	0.001
Non-immigrant	97.0%	97.0%	0.141	96.5%	96.5%	0.001
**Type of Lung Cancer**						
NSCLC: Adenocarcinoma	42.2%	35.5%	0.139	37.5%	36.8%	0.014
NSCLC: Squamous cell carcinoma	20.5%	21.9%	0.414	20.9%	21.6%	0.017
NSCLC: Large cell carcinoma	2.6%	2.4%	0.008	2.6%	2.5%	0.012
NSCLC: Adenosquamous	0.7%	0.5%	0.021	0.6%	0.6%	0.004
NSCLC: Not otherwise specified	13.6%	14.2%	0.017	14.0%	14.1%	0.001
SCLC	11.7%	12.7%	0.032	12.4%	12.5%	0.003
Unspecified	8.8%	12.7%	0.119	12.0%	12.0%	0.001
**Comorbidities**						
Asthma	9.9%	26.1%	0.385	22.7%	23.1%	0.009
Congestive heart failure	9.7%	17.7%	0.217	16.2%	16.2%	0
Dementia	2.8%	5.5%	0.125	5.3%	5.0%	0.012
Diabetes	23.3%	29.4%	0.136	29.0%	28.4%	0.015
Previous pneumonia	24.3%	32.5%	0.177	30.0%	30.9%	0.019
Cancer in the previous 5 years	6.6%	8.7%	0.074	8.6%	8.3%	0.010
**Previous Healthcare Utilization**						
Rate of primary care visits per year						
5 or more	31.3%	50.6%	0.386	47.4%	47.1%	0.007
Between 2 and 5	37.0%	35.1%	0.041	35.4%	35.5%	0.001
2 or less	24.9%	12.6%	0.346	14.7%	14.9%	0.007
0	6.7%	1.7%	0.312	2.5%	2.6%	0.004
Specialist care	34.7%	49.7%	0.301	47.8%	47.0%	0.015

Abbreviations: NSCLC = Non-small cell lung cancer, SCLC = small cell lung cancer, SMD = standardized mean difference, SD=standard deviation

Table S5: Sensitivity Analysis - Balance in characteristics of individuals with COPD and without COPD prior to and after inverse propensity score weighting (IPW)

	**Unweighted**			**Weighted**		
	**COPD** (n=25,941)	**No COPD** (n=52,051)	**SMD**	**COPD**	**No COPD**	**SMD**
**Demographics**						
Age, mean (SD)	72.6 (9.0)	69.3 (11.0)	0.316	71.0 (9.1)	70.6 (11.0)	0.044
Sex, % male	51.6%	51.0%	0.010	52.1%	51.3%	0.015
Rurality/Income Quintile						
Rural	16.3%	14.6%	0.049	15.3%	15.1%	0.005
Urban 1 (lowest)	24.5%	18.9%	0.138	20.9%	20.9%	0.001
Urban 2	19.7%	19.0%	0.018	19.3%	19.2%	0.002
Urban 3	15.8%	16.8%	0.026	16.4%	16.4%	0.001
Urban 4	13.0%	15.9%	0.083	14.8%	14.9%	0.004
Urban 5 (highest)	10.8%	14.9%	0.121	13.4%	13.5%	0.004
Immigration Category						
</= 10 years	0.3%	1.7%	0.126	1.0%	1.3%	0.021
Long-term resident (> 10 years)	2.3%	5.8%	0.168	4.4%	4.6%	0.013
Non-immigrant	97.4%	92.4%	0.210	94.6%	94.1%	0.022
**Type of Lung Cancer**						
NSCLC: Adenocarcinoma	33.0%	47.2%	0.288	42.6%	42.6%	0.001
NSCLC: Squamous cell carcinoma	23.4%	15.1%	0.215	18.0%	17.9%	0.003
NSCLC: Large cell carcinoma	2.3%	2.7%	0.023	2.6%	2.6%	0.002
NSCLC: Adenosquamous	0.5%	0.6%	0.016	0.6%	0.6%	0
NSCLC: Not otherwise specified	14.1%	14.2%	0.002	13.9%	14.1%	0.005
SCLC	12.4%	11.1%	0.039	11.4%	11.5%	0.003
Unspecified	14.3%	9.0%	0.172	10.9%	10.8%	0.004
**Comorbidities**						
Asthma	31.0%	10.0%	0.559	17.0%	16.8%	0.006
Congestive heart failure	21.5%	8.1%	0.404	12.9%	12.7%	0.005
Dementia	6.1%	3.8%	0.107	4.8%	4.7%	0.009
Diabetes	30.7%	25.3%	0.121	27.7%	27.1%	0.014
Previous pneumonia	37.4%	19.5%	0.413	25.6%	25.3%	0.005
Cancer in the previous 5 years	8.5%	9.1%	0.022	9.1%	8.9%	0.006
**Previous Healthcare Utilization**						
Rate of primary care visits per year						
5 or more	54.5%	36.2%	0.370	43.0%	42.4%	0.013
Between 2 and 5	33.2%	38.3%	0.104	37.0%	36.7%	0.006
2 or less	10.7%	20.8%	0.267	16.8%	17.3%	0.013
0	1.6%	4.7%	0.165	3.2%	3.6%	0.022
Specialist care	53.1%	38.0%	0.305	44.1%	43.2%	0.018

Abbreviations: NSCLC = Non-small cell lung cancer, SCLC = small cell lung cancer, SMD = standardized mean difference, SD=standard deviation
